# The c-di-AMP binding protein NadD from *Mesomycoplasma ovipneumoniae* functions as a phosphodiesterase that inhibits host inflammatory responses

**DOI:** 10.1186/s13567-025-01707-5

**Published:** 2026-01-09

**Authors:** Ying Zhang, Zhaokun Xu, Min Li, Xiujing Hao

**Affiliations:** 1 School of Life Science, Ningxia University, Yinchuan, China; 2 Institute of Medical Sciences, General Hospital of Ningxia Medical University, Yinchuan, China; 3 Key Lab of the Ministry of Education for Protection and Utilization of Special Biological Resources in Western China, Ningxia University, Yinchuan, China; 4 Ningxia Vocational and Technical University, Yinchuan, China

**Keywords:** *Mesomycoplasma ovipneumoniae*, sheep alveolar macrophages, c-di-AMP, phosphodiesterase, NadD

## Abstract

Cyclic dimeric adenosine monophosphate (c-di-AMP) is a second messenger commonly found in bacteria, particularly within the Firmicutes and Actinobacteria phyla, as well as in Archaea. Phosphodiesterase (PDE) is an enzyme that plays a crucial role in regulating the homeostasis of bacterial c-di-AMP and is capable of hydrolysing this molecule both intracellularly and extracellularly. This enzymatic activity influences various physiological functions in bacteria, modulates the host immune response, and impacts the pathogenic processes of infectious agents. *Mesomycoplasma ovipneumoniae* (*M. ovipneumoniae*) is the primary pathogen responsible for mycoplasma pneumonia in sheep, but its pathogenic mechanisms remain poorly understood. This study aimed to investigate the potential role of c-di-AMP and its binding proteins in *M. ovipneumoniae* in affecting host immune responses. Our findings confirmed that *M. ovipneumoniae* produced c-di-AMP in both the intracellular extracts and culture supernatants and that it generated c-di-AMP during the infection of sheep primary alveolar macrophages. Transcriptome analysis revealed that c-di-AMP can activate the immune response in macrophages. Furthermore, we identified and characterized the c-di-AMP binding protein nicotinate-nucleotide adenylyltransferase (NadD), which represents a novel class of PDE containing His-Asp (HD) domains in *Mycoplasmas*. Notably, our study demonstrated that NadD suppresses inflammatory responses in host cells infected with *M. ovipneumoniae*. In conclusion, this study provides new insights into the pathogenic mechanisms of *M. ovipneumoniae* and develops potential targets for the prevention and control of ovine mycoplasma pneumonia, as well as for the development of therapeutic agents.

**Supplementary Information:**

The online version contains supplementary material available at 10.1186/s13567-025-01707-5.

## Introduction

*Mesomycoplasma ovipneumoniae* (*M. ovipneumoniae*) is the primary pathogen responsible for ovine mycoplasma pneumonia [1]. Infection with *M. ovipneumoniae* can lead to an increased respiratory rate, coughing, runny nose, fever, progressive weight loss, and proliferative inflammation of the lung interstitium in sheep [2]. The incidence rate among sheep infected with *M. ovipneumoniae* ranges from 20 to 30%, with some regions reporting rates as high as 60% to 80%, resulting in significant economic losses for the sheep breeding industry [3]. Currently, the pathogenic mechanisms of *M. ovipneumoniae* remain unclear, which hinders the development of effective prevention and treatment strategies for ovine mycoplasma pneumonia. Existing research indicates that macrophages play a crucial role in the process of *M. ovipneumoniae* infection. *M. ovipneumoniae* primarily induces lesions in the trachea, bronchi, and lungs, which are characterized by necrosis and proliferation of tracheal mucosal epithelial cells, significant infiltration of inflammatory cells within the alveolar cavity, and an increase in pulmonary macrophages [1]. *M. ovipneumoniae* infection enhances both the transcription and translation of proinflammatory cytokine genes, including interleukin-1β (IL-1β), IL-18, and tumor necrosis factor-α (TNF-α), in a manner dependent on caspase-8, p53, and reactive oxygen species (ROS) [4]. These findings indicate that macrophages may play a critical role in the pathogenesis of *M. ovipneumoniae* infection. Nonetheless, the key factors and pathogenic mechanisms underlying the *M. ovipneumoniae*-induced macrophage immune response remain unclear.

Cyclic dimeric adenosine monophosphate (c-di-AMP) is a bacterial second messenger molecule found in prokaryotes, including bacteria [5], archaea [6], *Mycoplasmas* [7, 8], and *chlamydia* [9]. It plays a crucial role in various physiological processes in bacteria, such as growth, biofilm formation, virulence, and resistance [10, 11]. Additionally, c-di-AMP is involved in the pathogenicity of numerous pathogens and modulates the host immune response [12, 13]. During infection, c-di-AMP can be secreted into the cytoplasm of host cells by pathogens. This molecule activates the host cell immune response through pattern recognition receptors, notably by engaging the STING-TBK1-IRF3 pathway to induce an IFN-β response. Simultaneously, it facilitates interactions between pathogens and host cells by promoting the expression of a wide array of inflammatory factors [14]. *Mycoplasmas* are recognized as the smallest and simplest microorganisms capable of independent life [15]. However, to date, no studies on c-di-AMP in *M. ovipneumoniae* have been reported.

The metabolic level of c-di-AMP significantly influences the progression of microbial infections [16]. Phosphodiesterase (PDE) is an enzyme that regulates c-di-AMP levels in bacteria [17, 18]. Early studies on c-di-AMP-specific PDEs revealed that these enzymes possess a characteristic DHH/DHHA1 (Asp-His-His) domain or an HD (His-Asp) domain, which enables them to hydrolyse c-di-AMP into linear phosphoadenylyl adenosine (pApA) molecules or two molecules of AMP [19–21]. PDEs play crucial roles in directing innate immunity and mediating host‒pathogen interactions during infections [22]. The CdnP protein acts as a nucleotidase that hydrolyses c-di-AMP in the extracellular space. In *Mycobacterium tuberculosis*, CdnP inhibits innate immune cytosolic surveillance [23]. In *group B Streptococcus*, CdnP degrades cyclic di-AMP to modulate STING-dependent type I interferon production in macrophages [24].

This study demonstrated that *M. ovipneumoniae* can produce c-di-AMP signalling molecules both in vitro and during infection of host cells. Furthermore, c-di-AMP can activate the expression of key proteins and inflammatory factors within signalling pathways such as STING in sheep alveolar macrophages. This activation may play a crucial role in initiating the host immune response during *M. ovipneumoniae* infection of these macrophages. Our research also confirmed that the c-di-AMP binding protein NadD in *M. ovipneumoniae* possesses enzymatic activity that hydrolyses c-di-AMP. NadD can reduce the transcription levels of *IL-6*, *TNF-α*, and *IFN-β* in sheep alveolar macrophages infected with *M. ovipneumoniae*, thereby mitigating the inflammatory response of the cells. Our study enhances the understanding of the metabolic pathway of the second messenger c-di-AMP in *M. ovipneumoniae*, elucidates its role in the interaction between sheep alveolar macrophages and *M. ovipneumoniae*, and offers new insights into the pathogenesis of *M. ovipneumoniae*.

## Materials and methods

### *M. ovipneumoniae* culture conditions and growth curves

The original culture of the *M. ovipneumoniae* strain Y98 was preserved at the Key Laboratory of the Ministry of Education for Protection and Utilization of Special Biological Resources in Western China and was stored frozen at − 70 °C. As previously described [25, 26], the *M. ovipneumoniae* bacteria were removed and resuscitated in PPLO broth at 37 °C, followed by culture expansion and determination of color change units (CCU). *E. coli* strains DH5α and BL21 (DE3) (TransGen Biotech, Beijing, China) were cultured in Luria–Bertani (LB) broth at 37 °C and utilized for DNA cloning and protein expression, respectively.

### Quantification of secreted and intracellular c-di-AMP of *M. ovipneumoniae*

C-di-AMP was extracted from *M. ovipneumoniae* as previously described [8]. Briefly, *M. ovipneumoniae* cells were cultivated in 300 mL of PPLO medium, and the culture supernatant and bacterial pellet were collected separately. The precipitate was extracted using an extraction mixture (acetonitrile/methanol/water 40/40/20 v/v/v). The extract was then dried under high-speed vacuum for 2 h and subsequently redissolved in deionized water for detection.

Commercial c-di-AMP (MCE, USA) was utilized as the standard, and the concentration of c-di-AMP in *M. ovipneumoniae* was determined via UPLC-MS, as previously described [27]. A 20 μL sample was subjected to reverse-phase UPLC with an RPC-18 column (1.8 μm, 250 × 4.5 mm) equilibrated with 95% 20 mM ammonium acetate (pH 8.0) and 5% ethanol. The same buffer was used in the elution program at a flow rate of 0.7 mL/min. Nucleotides were monitored at 254 nm. The c-di-AMP in *M. ovipneumoniae* was subsequently detected with a triple-quadruple mass spectrometer equipped with an electrospray ionization source via multiple reaction monitoring transitions of m/z 657 → 124 in negative ionization mode.

### Isolation and identification of primary sheep alveolar macrophages

Sheep lungs aged 3 to 6 months were obtained from the Yongning slaughterhouse in Yinchuan, Ningxia. Sterile phosphate-buffered saline (PBS), containing a 5% solution of penicillin‒streptomycin-amphotericin B, was perfused into the lungs until they were fully inflated. The lungs were then gently rubbed for 5 min, after which the lavage fluid was collected. This lavage procedure was repeated three times. The collected lavage fluid was centrifuged at 800 × *g* for 5 min to isolate the cells, which were then resuspended in PBS, filtered through a 75 μm filter, and subjected to a second centrifugation to further collect the cells. If the isolated cells contained a significant number of blood cells, density gradient purification was performed. The purified cells were centrifuged at 800 × *g* for 5 min, resuspended in RPMI 1640 medium (Gibco, USA) supplemented with 15% heat-inactivated fetal bovine serum (FBS) (Gibco, USA) and 1% penicillin‒streptomycin, seeded into the cell plates, and then washed with PBS 2 h later. The cells were cultured at 37 °C in a humidified atmosphere containing 5% CO_2_. The isolated and cultured primary sheep alveolar macrophages were identified by detecting the macrophage-specific surface antigen CD14 (ab186689; Abcam; UK) via flow cytometry and immunofluorescence. The study protocol was reviewed and approved by the Animal Welfare & Ethics Committee of Ningxia University (Ningxia, China) (No. NXU-2024–161).

### *M. ovipneumoniae* intracellular infection and monitoring

Prior to infection, bacteria were collected, washed in PBS, and resuspended in cell culture media. A multiplicity of infection (MOI) of 15 was used to infect sheep alveolar macrophages. At 0, 3, 6, 12, and 24 h, the supernatant was removed, and the cells were washed three times with cell culture media. On the one hand, the *M. ovipneumoniae* marker gene HSP70 was amplified by PCR to monitor *M. ovipneumoniae* in sheep alveolar macrophages. On the other hand, prior to conducting the coculture experiment of *M. ovipneumoniae* and sheep alveolar macrophages, the *M. ovipneumoniae* cell membrane was stained with 10 μM Dio dye. A control group was also set up, in which PBS was mixed with 10 μM DiO dye and incubated for the same duration to eliminate the possibility of nonspecific staining by the dye. After incubation, the cells were washed multiple times with PBS to remove any *M. ovipneumoniae* that had not adhered to the surface of the sheep alveolar macrophages or had not been internalized by them. Next, TRITC-labelled phalloidin was used to stain the cytoskeleton of the sheep alveolar macrophages, and DAPI was used to label the cell nuclei. Finally, the infection process of *M. ovipneumoniae* in sheep alveolar macrophages was observed using a laser confocal microscope. In addition, the concentration of c-di-AMP in sheep alveolar macrophages infected with *M. ovipneumoniae* was determined using HPLC, as previously described [8].

### RNA isolation, transcriptomics, and RT‒qPCR

After sheep alveolar macrophages were treated with *M. ovipneumoniae* and c-di-AMP, total RNA was extracted using TRIzol reagent (Invitrogen, USA). Sequencing was performed on the Illumina NovaSeq™ 6000 platform provided by Baiqu Biomedical Technology Co., Ltd. (Shanghai, China). The expression abundance of mRNA was quantified by calculating the fragments per kilobase of transcript per million mapped reads (FPKM) values. Differential expression analysis of genes between the two groups was conducted using DESeq2 and edgeR software. Reverse transcription was executed using PrimeScript™ RT Master Mix (Takara, Japan), followed by real-time PCR with TB Green® Premix Ex Taq™ (Takara, Japan) to validate gene mRNA expression. The sequences of the real-time PCR primers were synthesized by Sangon Biotech (Shanghai, China). Relative mRNA expression was normalized to that of GAPDH and calculated using the 2^−ΔΔCt^ method. Additional file 1 shows the primer sequences.

### Pull-down of c-di-AMP-binding proteins via c-di-AMP-coupled agarose

The *M. ovipneumoniae* bacterial pellet was collected and incubated in pull-down buffer (100 mM Tris–HCl, pH 7.5; 100 mM KCl; 150 mM NaCl; 5 mM MgCl2; 0.5 mM DTT; 0.1% Tween-20; 1 × protease inhibitor), and the cells were disrupted at 30 Hz by sonication for 2 × 2.5 min, followed by incubation on ice. The mixture was centrifuged at 12 000 × *g* for 10 min at 4 °C, and the supernatant was carefully aspirated. Two hundred microlitres of 2'-AHC-c-diAMP-agarose (BIOLOG, Germany) or EtOH-NH agarose (BIOLOG, Germany) were equilibrated three times with 1 mL of pull-down buffer. After equilibration, 500 μL of the cell lysate was incubated with the agarose matrix overnight at 4 °C with rotation. The supernatant was discarded, and the matrix was washed three times with PBS. The bound material was eluted with 200 μL of 1 × PBS (pH 7.4, containing 1 mM c-di-AMP) and incubated at room temperature for 20 min. The sample was then centrifuged at 2000 × *g* for 15 min at 4 °C, after which the supernatant was carefully aspirated. The sample was precipitated with acetone at -20 °C overnight. The following day, the sample was centrifuged at 14 800 × *g* for 1 h at 4 °C, and the precipitate was dried at room temperature for 15 min. The remaining pellet was resuspended in 40 mL of PBS and subsequently analysed using SDS‒PAGE. Proteins of interest were identified through mass spectrometry.

### Protein cloning, expression, and purification

The NadD gene sequence was codon optimized and cloned and inserted into the pET-28b vector. This vector was then transformed into the *E. coli* BL21 (DE3) strain, and the cells were induced with isopropyl β-D-1-thiogalactopyranoside (IPTG). The harvested cells were suspended in buffer containing 50 mM phosphate (pH 7.6), 500 mM NaCl, and 10 mM imidazole, followed by lysis through sonication. The cell lysates were centrifuged for 30 min at 4 ℃ and purified using an ÄKTA pure (Cytiva, USA) equipped with a HiTrap TALON crude (Cytiva, USA). Protein samples were quantified using the BCA assay and subsequently stored at -80 ℃.

### Surface plasmon resonance

The Biacore T200 system (Cytiva, USA) was used to detect the binding of c-di-AMP to the NadD protein. The Series S Sensor Chip CM5 (Cytiva, USA) was activated using 400 mM 1-(3-dimethylaminopropyl)-3-ethylcarbodiimide (EDC) and 100 mM N-hydroxysuccinimide (NHS) solutions. The NadD recombinant protein was then immobilized on the chip, and various concentrations of c-di-AMP (MCE, USA) were analysed via a multicycle method. Upon completion of the run, Biacore T200 evaluation software (Cytiva, USA) was used to assess the binding affinity between c-di-AMP and the NadD recombinant protein.

### Phosphodiesterase activity assay

The reaction buffer consisted of 100 mM Tris–HCl (pH 7.5), 20 mM KCl, 2 mM CoCl_2_, and 50 μM c-di-AMP. The reaction was initiated by adding 5 μM NadD recombinant protein to 100 μL of the reaction buffer, followed by incubation for 2 h at 37 °C. At the designated time points, the samples were heated to 95 °C for 5 min, centrifuged at maximum speed to remove the precipitate, and analysed by UPLC/MS for c-di-AMP degradation. For pH dependence determination, reversed-phase HPLC was conducted using a reaction buffer containing 2 mM CoCl_2_ under 100 mM Tris–HCl at pH 6.8, 7.5, 8.0, 8.5, and 8.8. The metal ion specificity of NadD was determined by monitoring the activity of the NadD enzyme in the presence of 2 mM CoCl_2_, MnCl_2_, NiCl_2_, FeCl_2_, FeCl_3_, or MgCl_2_. The relative activity of NadD under these different conditions was calculated on the basis of the peak area of AMP generated. The c-di-AMP standard was purchased from MCE, the 5'-pApA standard was purchased from BioLog, and the AMP standard was purchased from Sigma.

### Western blot

Equal amounts of protein were resolved by SDS‒PAGE and subsequently transferred to PVDF membranes (Millipore, USA). After being blocked with 5% skim milk, the membranes were incubated overnight at 4 °C with a primary antibody diluted according to the manufacturer's instructions. GAPDH (Proteintech, China) was used as an internal control. Following washing with TBST, the membranes were incubated for 1 h with an HRP-conjugated secondary antibody (Proteintech), followed by another wash with TBST. Signal detection was performed via an enhanced chemiluminescence (ECL) detection system.

### Statistical analysis

All experiments were performed in triplicate, and the results were obtained from three independent experiments. The data are expressed as the means ± standard deviations (X ± SD). Comparisons between two groups were conducted by unpaired t tests, and comparisons among multiple groups were conducted by one-way ANOVA. *, **, and *** represent *p* < 0.05, *p* < 0.01, and *p* < 0.001, respectively.

## Results

### C-di-AMP production during *M. ovipneumoniae* infection of sheep alveolar macrophages

**Figure 1:**
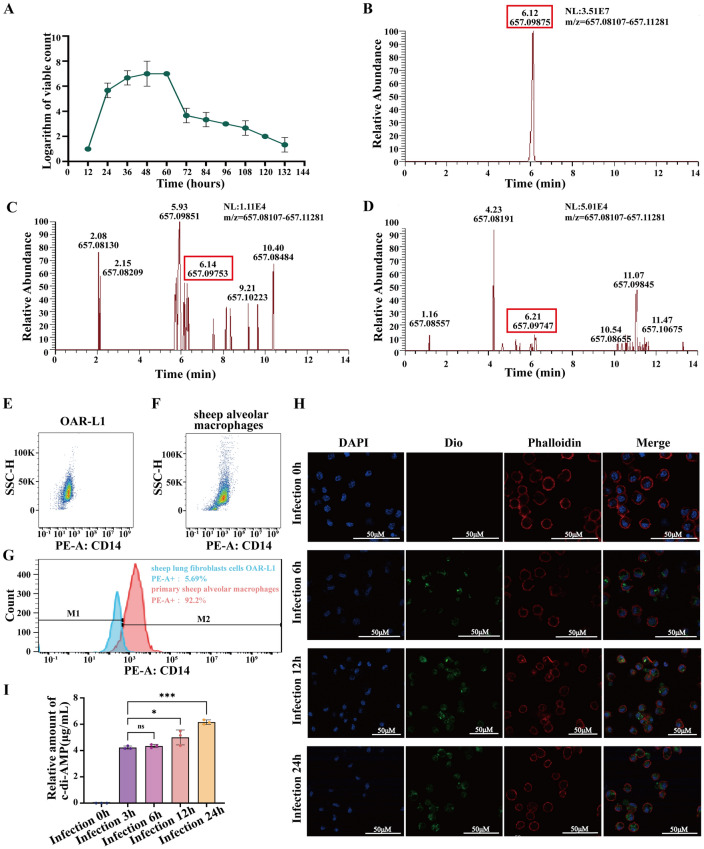
**c-di-AMP production during** ***M. ovipneumoniae *infection of sheep alveolar macrophages**. **A** Growth curve of *M. ovipneumoniae*. **B–D** ESI‒MS analysis of relevant HPLC fractions from *M. ovipneumoniae* cellular extracts and culture supernatants; **B** c-di-AMP standard; **C** *M. ovipneumoniae* intracellular extracts; **D** *M. ovipneumoniae* culture supernatants. **E–G** Flow cytometry was used to detect the specific surface antigen CD14 on primary sheep alveolar macrophages, with OAR-L1 sheep lung fibroblasts used as a control. **E** Flow cytometric collection diagram for OAR-L1 sheep lung fibroblasts. **F** Flow cytometric collection diagram for primary sheep alveolar macrophages. **G** Flow cytometric analysis diagram of OAR-L1 sheep lung fibroblasts and primary alveolar macrophages. M1 indicates the fluorescently labelled negative interval, whereas M2 represents the fluorescently labelled positive interval. **H** Fluorescence observation of *M. ovipneumoniae*-infected sheep alveolar macrophages. Blue: DAPI; green: DiO-labelled *M. ovipneumoniae*; red: phalloidin-labelled cytoskeleton. **I** Quantitative analysis of intracellular c-di-AMP during *M. ovipneumoniae* infection of sheep alveolar macrophages. The means ± SDs of 3 independent experiments were compared by one-way ANOVA.

To determine whether *M. ovipneumoniae* produces c-di-AMP, we first measured the growth curve of *M. ovipneumoniae* using CCU. The strain exhibited exponential growth from 12 to 48 h post-inoculation, followed by a stable phase from 48 to 60 h, after which the number of viable bacteria began to decrease (Figure 1A). We subsequently employed a commercial c-di-AMP standard as a control and collected intracellular extracts and culture supernatants of *M. ovipneumoniae* during the exponential growth phase for UPLC‒MS analysis. We observed a peak at approximately 6.21 min, which was identical to the c-di-AMP standard, in both the intracellular extract and the culture supernatant of *M. ovipneumoniae*. This peak exhibited the same molecular fragment as the c-di-AMP standard, indicating the presence of c-di-AMP in *M. ovipneumoniae* and suggesting that this signalling molecule can be secreted outside the cell (Figure 1B–D).

To further investigate whether *M. ovipneumoniae* can also produce c-di-AMP during infection of primary sheep alveolar macrophages, we first confirmed the reliability of the isolated sheep alveolar macrophages by identifying the macrophage-specific surface antigen CD14 in the sheep alveolar lavage fluid. The flow cytometry results revealed that 92.2% of the cells in the sheep bronchoalveolar lavage fluid expressed the macrophage-specific surface antigen CD14, in contrast to the sheep lung fibroblasts (OAR-L1) (Figure 1E‒G). The immunofluorescence results revealed that the isolated sheep bronchoalveolar lavage cells were labelled with an anti-CD14 antibody and exhibited green fluorescence, whereas the OAR-L1 cells did not display green fluorescence (Additional file 2A). Similarly, PCR and fluorescence tracking of *M. ovipneumoniae* infection in primary sheep alveolar macrophages revealed that the *M. ovipneumoniae*-specific marker gene Hsp70 was detectable in samples of infected macrophages at 6 h, 12 h, and 24 h post infection (Additional file 2B). Observations via laser confocal microscopy revealed that, in comparison with those in the PBS control group, green fluorescent-labelled *M. ovipneumoniae* were attached to both the surface and interior of the sheep alveolar macrophages after coculture for 6 h, 12 h, or 24 h. (Figure 1H, Additional file 2C). Furthermore, commercial c-di-AMP was utilized as the reference standard. Following *M. ovipneumoniae* infection of primary sheep alveolar macrophages, cell lysates were collected, and the c-di-AMP content within these macrophages was analysed via HPLC after freezing. As expected, c-di-AMP was detectable in the cells three hours after *M. ovipneumoniae* infection, and the intracellular c-di-AMP content progressively increased with increasing infection duration. (Figure 1I, Additional file 2D–I).

### C-di-AMP has the ability to activate the immune response in primary sheep alveolar macrophages

**Figure 2:**
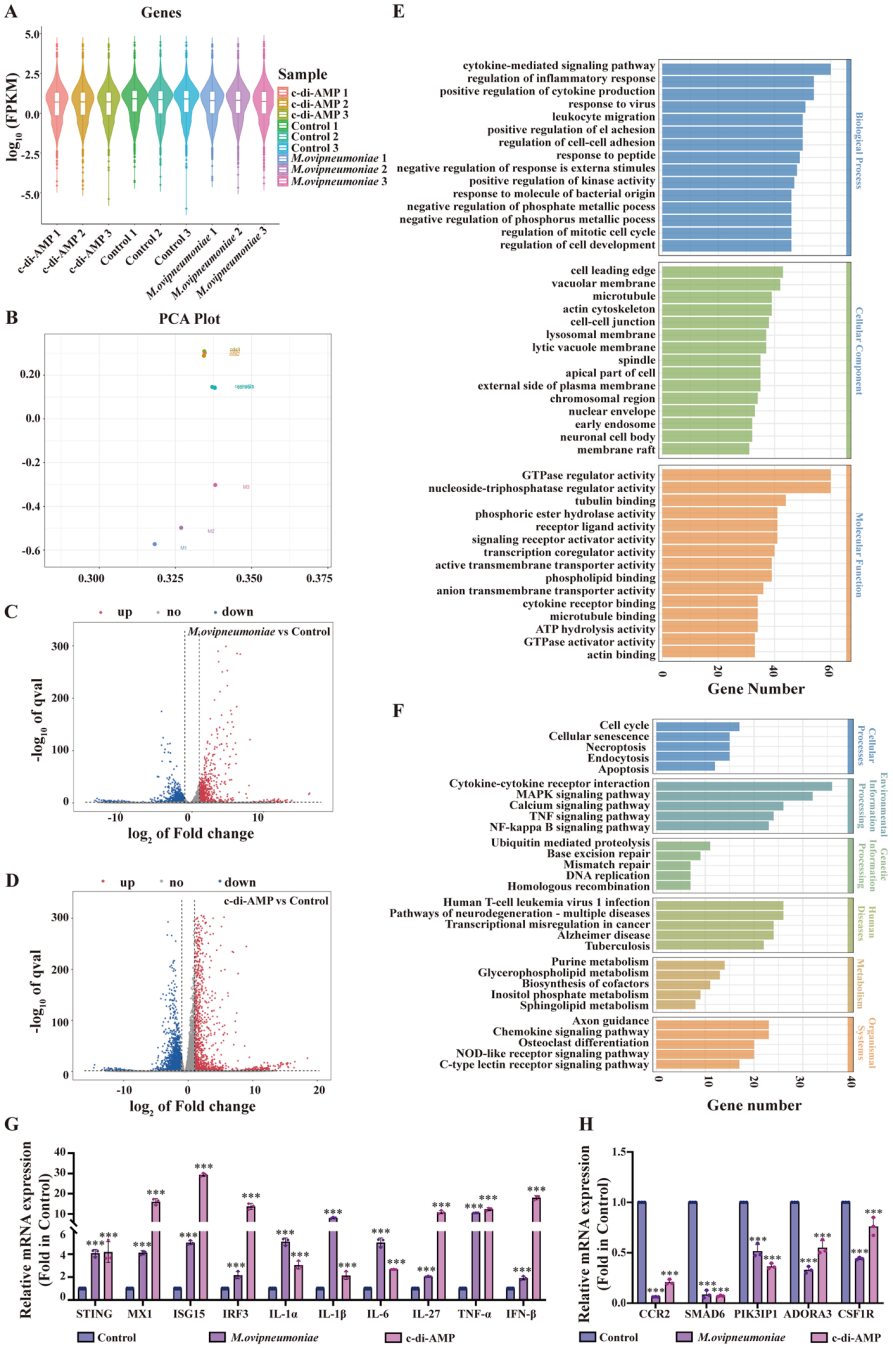
**Transcriptomic analyses reveal that c-di-AMP activates immune responses in primary sheep alveolar macrophages**. **A** The FPKM violin plot displays gene expression across each sample. **B** PCA of the samples is presented. **C** A volcano plot highlights the differentially expressed genes in the *M. ovipneumoniae* group versus the control group. **D** Volcano plot showing the differentially expressed genes in the c-di-AMP group versus the control group. **E** GO enrichment analysis identified genes whose expression was altered in the *M. ovipneumoniae* and c-di-AMP groups compared with the control group. **F** KEGG enrichment analysis of the genes whose expression changed in the *M. ovipneumoniae* and c-di-AMP groups relative to that in the control group. **G** RT‒qPCR verification of differentially upregulated genes in the transcriptome. **H** RT‒qPCR verification of differentially downregulated genes in the transcriptome. The means ± SDs of 3 independent experiments were compared by one-way ANOVA. Statistical significance is indicated as follows: * *p* < 0.05, ** *p* < 0.01, *** *p* < 0.001, ns: not significant.

During the infection process, various components of pathogenic bacteria can trigger the host immune response. As a second messenger of bacteria, c-di-AMP plays a crucial role in this context [22]. To analyse the function of c-di-AMP during *M. ovipneumoniae* infection of sheep alveolar macrophages, we conducted transcriptomic analyses of *M. ovipneumoniae*-infected and c-di-AMP-treated sheep alveolar macrophages, with untreated sheep alveolar macrophages used as a control. Violin plots and PCA consistently demonstrated a strong correlation and repeatability among the samples. Notably, PCA revealed that c-di-AMP-treated sheep alveolar macrophages resemble untreated sheep alveolar macrophages more closely than do *M. ovipneumoniae*-treated sheep alveolar macrophages (Figures 2A, B). Compared with the control group, the c-di-AMP group presented the upregulation of 1499 genes and the downregulation of 3010 genes, whereas the *M. ovipneumoniae* group presented the upregulation of 723 genes and the downregulation of 1647 genes (Figures 2C, D). The DEGs identified in both the *M. ovipneumoniae* and c-di-AMP groups were associated primarily with pathways related to cytokine-mediated signalling, the regulation of the inflammatory response, the positive regulation of cytokine production, GTPase regulator activity, nucleoside-triphosphatase regulator activity and phosphoric ester hydrolase activity. Additionally, these genes are involved in signalling pathways such as necroptosis, cytokine‒cytokine receptor interactions, the MAPK signalling pathway, the TNF signalling pathway, and the NF‒kappa B signalling pathway (Figures 2E, F). We have included a list of genes and pathways that are similarly induced by *M. ovipneumoniae* and c-di-AMP in Additional file 3. Furthermore, several key genes and inflammatory factors within the signalling pathway were selected for mRNA expression analysis via RT‒qPCR, and the results were consistent with the transcriptome data (Figures 2G, H). These findings suggest that c-di-AMP may play a significant role in the *M. ovipneumoniae*-induced immune response of sheep alveolar macrophages.

### C-di-AMP can specifically bind to the NadD protein of *M. ovipneumoniae*

**Figure 3:**
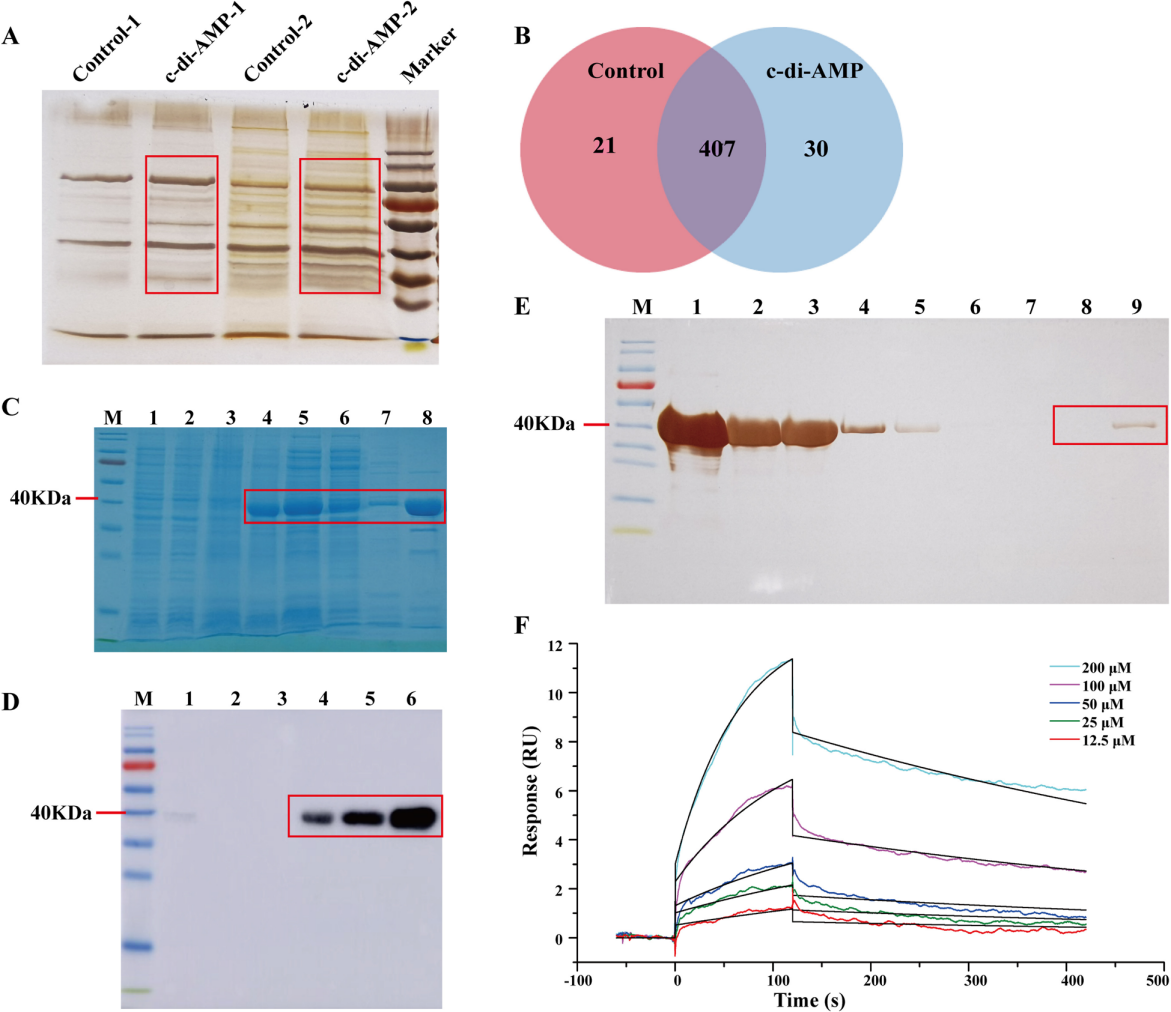
**Specific binding of c-di-AMP to the NadD protein of** ***M. ovipneumoniae***. **A** The c-di-AMP-binding proteins were identified by silver staining. Control: agarose coupled with ethanolamine; c-di-AMP: agarose coupled with c-di-AMP. Silver staining was performed in 2 independent experiments. **B** A Venn diagram depicting the identification of the spectrum of c-di-AMP-binding proteins. **C** The identification of the purified NadD protein by SDS‒PAGE analysis. Lane 1: the empty vector before induction; lane 2: the empty vector after 16 h of induction; lane 3: the pET-28b-NadD vector prior to induction; lane 4: the pET-28b-NadD vector after 16 h of induction; lane 5: the supernatant protein following the 16-h induction of the pET-28b-NadD vector; lane 6: the running-off liquid; lane 7: the washing solution; lane 8: the elution solution. **D** Western blot analysis confirmed the presence of the purified NadD protein. Lane 1: the empty vector before induction; lane 2: the empty vector after 16 h of induction; lane 3: the pET-28b-NadD vector before induction; lane 4: the pET-28b-NadD vector after 16 h of induction; lane 5: the supernatant protein after the 16-h induction of the pET-28b-NadD vector; lane 6: the elution mixture. **E** Silver staining results demonstrating the binding of the recombinant NadD protein to c-di-AMP. M: marker; lane 1: NadD recombinant protein; lane 2: running-off of the control group; lane 3: running-off of the c-di-AMP group; lane 4: washing solution II of the control group; lane 5: washing solution II of the c-di-AMP group; lane 6: washing solution III of the control group; lane 7: washing solution III of the c-di-AMP group; lane 8: eluate of the control group; lane 9: eluate of the c-di-AMP group. **F** The concentration gradient binding curve between NadD and c-di-AMP is shown, as detected by SPR.

To identify proteins that bind c-di-AMP, we used an affinity pull-down strategy with c-di-AMP–conjugated sepharose beads, which were incubated with *M. ovipneumoniae* cell lysate. Ethanolamine-conjugated beads served as a control to assess nonspecific binding to the sepharose scaffold. The silver staining results of the pulled-down proteins revealed significant differences in band intensity between the control group and the c-di-AMP group (Figure 3A). Mass spectrometry identified 30 unique proteins in the c-di-AMP group, while 246 proteins were found to be shared between the two groups, with higher expression levels in the c-di-AMP group (Figure 3B, Additional file 4). These proteins are associated primarily with cellular nitrogen compound metabolic processes, cellular components, and nucleic acid binding and are involved in biological processes such as RNA polymerase activity, nucleotide metabolism, and ribosome function (Additional file 5). Studies have shown that c-di-AMP in *Listeria monocytogenes* can specifically bind to its phosphodiesterase [21]. On this basis, we screened candidate proteins with phosphodiesterase functional domains that interact with c-di-AMP through protein conserved domain analysis. Among them, nicotinamide adenine dinucleotide (NadD) was more enriched in the c-di-AMP group and is closely related to the nucleotide metabolism process. Although NadD is not the protein with the most significant differential folding changes, it remains the focus of our research.

To further confirm the specific interaction between the NadD protein and c-di-AMP in *M. ovipneumoniae*, the NadD protein was expressed and purified via a prokaryotic expression system (Figures 3C, D). It was then incubated with agarose gel beads immobilized with c-di-AMP, while gel beads immobilized with ethanolamine served as a nonspecific binding control. The binding of the NadD protein was clearly detected in the c-di-AMP group (Figure 3E). Similarly, surface plasmon resonance (SPR) analysis confirmed the specific binding of c-di-AMP to the NadD protein. The fitting curve from the reaction between c-di-AMP and the NadD protein indicates that varying concentrations of c-di-AMP yield a concentration-dependent binding response value (RU) to the recombinant NadD protein, with an increase in response observed as the concentration of c-di-AMP increases (Figure 3F).

### NadD is a c-di-AMP phosphodiesterase in *M. ovipneumoniae*

**Figure 4:**
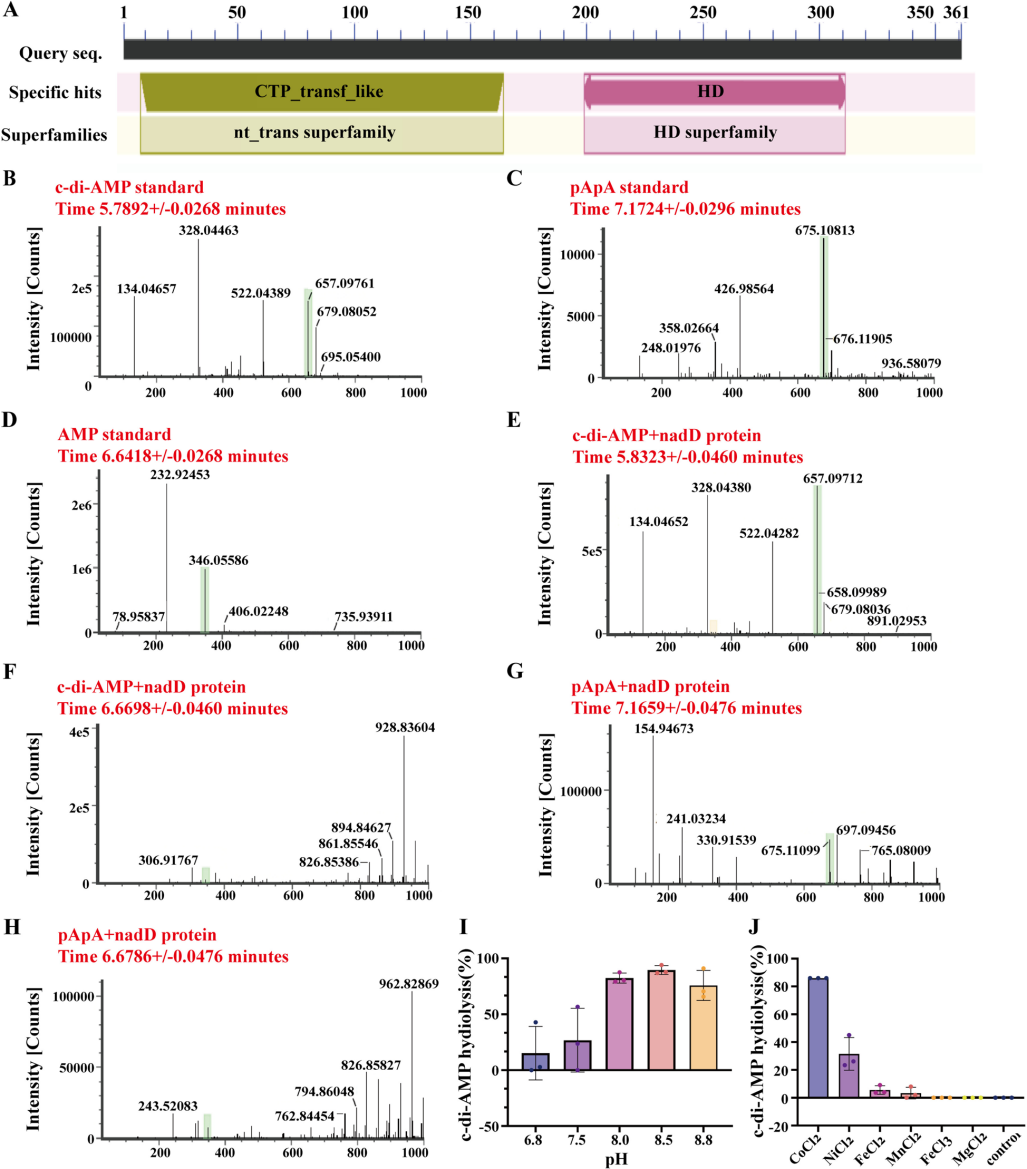
**Biochemical characterization of NadD as a phosphodiesterase**. **A** Analysis of the conserved domains of NadD. **B–H** LC‒MS analysis of the hydrolysis of c-di-AMP and pApA by NadD. **B**–**D** depict the standard, while **E**–**F** show the enzymatic reaction products of NadD with c-di-AMP, and G-H illustrate the enzymatic reaction products of NadD with pApA. **I** HPLC analysis of c-di-AMP hydrolysis by NadD at various pH values. **J** HPLC analysis of NadD-mediated hydrolysis of c-di-AMP in the presence of different metal ions. Three independent experiments were repeated by HPLC.

Preliminary analysis of the function of NadD, on the basis of its amino acid sequence, revealed that NadD is widely present in archaea, bacteria, and *Mycoplasma*, although it occupies a distinct branch within *Mycoplasma* (Additional file 6). NadD is classified as a nicotinamide nucleotide adenosyltransferase that belongs to the NadD superfamily. It is characterized by the presence of a conserved cytidylyltransferase-like (CTP_transf_like) domain and a novel superfamily of metal-dependent phosphohydrolases, known as the HD domain (Figure 4A). The amino acid sequence of the C-terminal HD domain of the NadD protein of *M. ovipneumoniae* shares low homology with bacterial HD domain-containing phosphodiesterases; however, these homologous proteins all possess a highly conserved HD (His-Asp) motif (Additional file 7). Given that the NadD protein of M. ovipneumoniae can bind to c-di-AMP and features the primary functional HD domain capable of hydrolysing c-di-AMP, it is hypothesized that the NadD protein of *M. ovipneumoniae* may have the potential to hydrolyse c-di-AMP. To further confirm whether the NadD protein can hydrolyse c-di-AMP, an in vitro enzymatic reaction system was established using c-di-AMP and pApA as substrates. UPLC-MS detection, which uses c-di-AMP, pApA, and AMP standards as references, confirmed that the NadD protein directly cleaves c-di-AMP or pApA into AMP in solution, indicating its potential role as a phosphodiesterase in *M. ovipneumoniae* (Fig. 4B–H). The catalytic activity of NadD is adaptable across a wide pH range (Figure 4I, Additional file 8), reaching its peak at pH 8.5. Additionally, the catalytic reaction of NadD can efficiently utilize Co^2+^ (Figure 4J, Additional file 9).

### NadD suppresses inflammatory responses in host cells infected by *M. ovipneumoniae*

**Figure 5:**
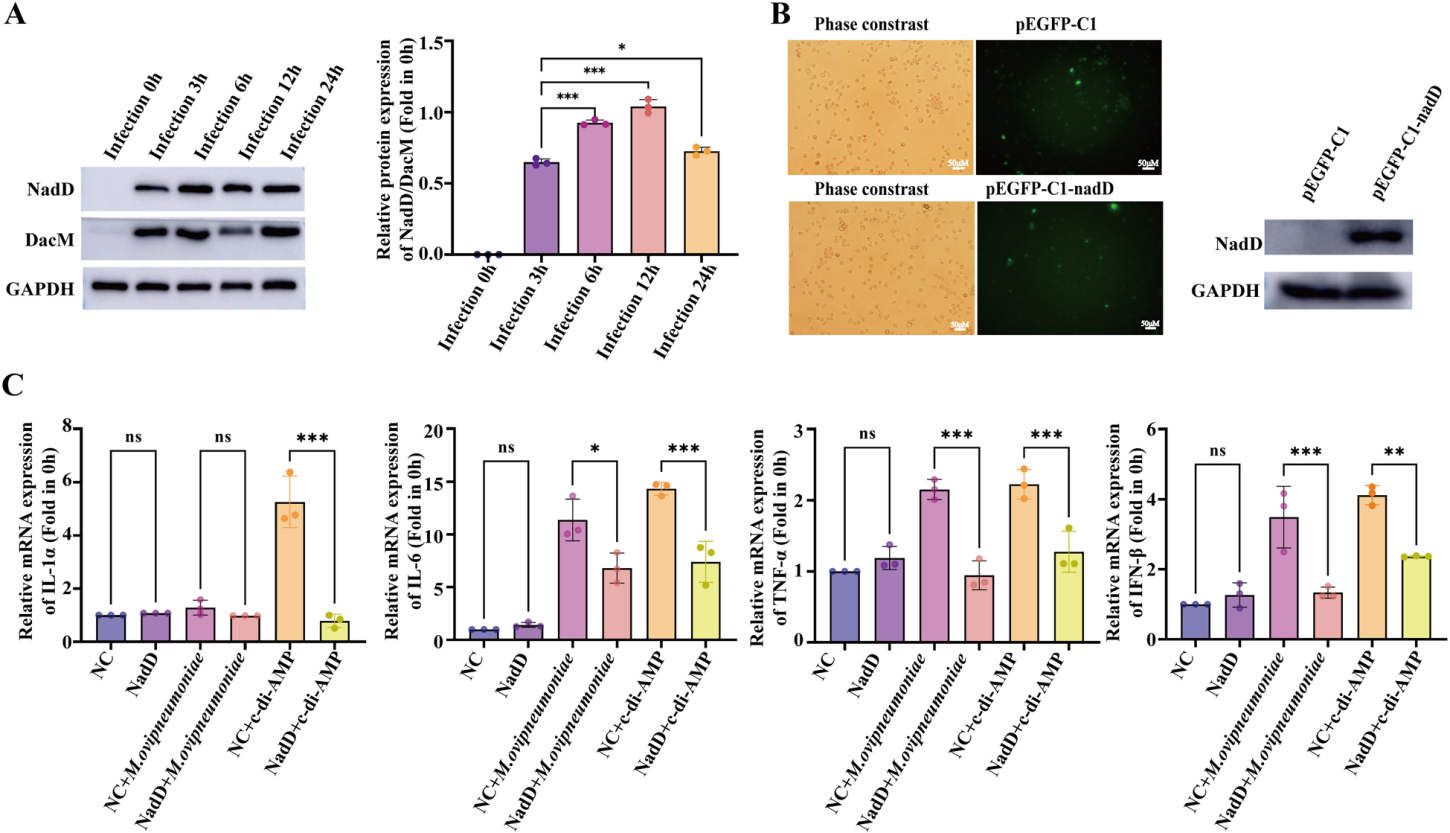
**NadD suppresses inflammatory responses in host cells infected with** ***M. ovipneumoniae***. **A** Western blot analysis and quantification of NadD and DacM proteins during *M. ovipneumoniae* infection of sheep alveolar macrophages. **B** Fluorescence observations (left) and western blot results (right) for the cells transfected with the pEGFP-C1 and pEGFP-C1-NadD plasmids. **C** RT‒qPCR analysis of the effect of the NadD protein on the mRNA expression of inflammatory factors during *M. ovipneumoniae* infection of sheep alveolar macrophages. NC + *M. ovipneumoniae*, NadD + *M. ovipneumoniae*, NC + c-di-AMP, and NadD + c-di-AMP represent the conditions where sheep alveolar macrophages were first transfected with an empty plasmid (NC) or the NadD plasmid, followed by infection with *M. ovipneumoniae* or treatment with c-di-AMP. The means ± SDs of 3 independent experiments were compared by one-way ANOVA. * *p* < 0.05, ** *p* < 0.01, *** *p* < 0.001, ns: not significant.

We detected the expression of the c-di-AMP phosphodiesterase NadD and the diadenylate cyclase DacM, a critical enzyme that catalyzes the synthesis of c-di-AMP, during the infection of sheep alveolar macrophages by *M. ovipneumoniae*. Compared with that at 3 h of infection, the ratio of NadD to DacM gradually increased at 6 and 12 h of infection, peaking at 12 h before beginning to decrease. These findings suggest that *M. ovipneumoniae* coordinately regulates the dynamic balance of c-di-AMP in bacteria through NadD and DacM during the infection of sheep alveolar macrophages. Such regulation may influence host inflammatory responses and modulate immune responses (Figure 5A). To preliminarily explore the function of the NadD protein during *M. ovipneumoniae* infection of sheep alveolar macrophages, we constructed the pEGFP-C1-NadD plasmid and transfected it into these cells. Fluorescence observation and western blot analysis confirmed the successful expression of the NadD protein in sheep alveolar macrophages (Figure 5B). Following the overexpression of the NadD protein, these cells were treated with *M. ovipneumoniae* or c-di-AMP. Compared with that in the control group, which received the empty plasmid (NC), the mRNA expression level of *IL-1α* in macrophages infected with *M. ovipneumoniae* after transfection with the NadD plasmid did not significantly change. However, the mRNA expression level of *IL-1α* in sheep alveolar macrophages transfected with the NadD plasmid and subsequently treated with c-di-AMP was significantly reduced. Additionally, in sheep alveolar macrophages transfected with the NadD plasmid and subsequently infected with *M. ovipneumoniae* or treated with c-di-AMP, the expression levels of *IL-6*, *TNF-α*, and *IFN-β* were also significantly downregulated (Figure 5C). These findings suggest that NadD may inhibit the expression of genes associated with cellular inflammatory factors by hydrolysing c-di-AMP, which is produced by bacteria during the infection of sheep alveolar macrophages by *M. ovipneumoniae*.

## Discussion

*M. ovipneumoniae* can cause mycoplasma pneumonia in sheep, particularly in lambs aged 1 to 3 months [28, 29]. However, the pathogenic mechanisms of *M. ovipneumoniae* remain unclear, and a systematic understanding of its virulence factors is lacking. Inflammatory injury in the lungs of sheep is a significant pathological feature following *M. ovipneumoniae* infection. *M. ovipneumoniae* infection can induce the upregulation of *IL-1β*, *IL-6*, *TNF-α*, and *NF-κB* gene expression in the trachea, bronchi, and lungs of sheep, thereby causing damage to tissues [30]. As innate immune cells, macrophages secrete a variety of active molecules, including ROS, NO, and inflammatory factors such as IFN-γ and IL-6, in response to pathogenic bacteria to combat infection [31]. Although studies have demonstrated that *M. ovipneumoniae*-derived lipid-associated membrane proteins (MLKs) can induce mouse peritoneal macrophages to secrete cytokines via TLR2 signalling [32], the components of *M. ovipneumoniae* that activate the host macrophage immune response remain unclear. Additionally, the species specificity and limitations of immortalized cell lines may hinder the accuracy of these findings in reflecting the actual infection scenario. Therefore, the use of primary host cells as an infection model may yield more valuable insights into the pathogenesis of *M. ovipneumoniae*. In this study, we isolated and cultured sheep alveolar macrophages from the lung tissue of sheep aged 3–6 months and established an *M. ovipneumoniae* infection model to explore the pathogenic mechanism of *M. ovipneumoniae*.

C-di-AMP is a second messenger molecule found in bacteria [5]. Host cells are capable of detecting c-di-AMP signals from bacteria, which activate the STING-TBK1-IRF3 signalling pathway, leading to the production of IFN-β in response to pathogenic bacterial infections [33]. Our experimental results demonstrate that *M. ovipneumoniae* can synthesize c-di-AMP during the infection of sheep alveolar macrophages, with the relative intracellular concentration of c-di-AMP progressively increasing as the duration of infection increases. Through transcriptome sequencing, we analysed the genes whose expression was altered in sheep alveolar macrophages during *M. ovipneumoniae* infection and c-di-AMP treatment. This analysis revealed that c-di-AMP may play a pivotal role in enabling *M. ovipneumoniae* to activate signalling pathways such as STING and to modulate the expression of inflammatory factors. C-di-AMP regulates various physiological processes through direct interactions with target proteins or by modulating their expression [34, 35]. Previous studies have employed affinity pull-down strategies in conjunction with proteomics technology to identify the PgpH protein of *Listeria monocytogenes* as a molecular target of c-di-AMP [21]. Similarly, we utilized an affinity pull-down strategy to screen for c-di-AMP-binding proteins in *M. ovipneumoniae* and discovered that c-di-AMP binds to the NadD protein of *M. ovipneumoniae*. This finding was further validated through SPR experiments. Through an analysis of the database from the Bacterial and Viral Bioinformatics Resource Center, we found that out of 170 *M. ovipneumoniae* strains collected globally, 159 strains possessed the NadD gene (Additional file 10). These findings suggest that the NadD gene is prevalent among various strains of *M. ovipneumoniae*. The NadD gene sequence utilized in this study was derived from the *M. ovipneumoniae* Y98 strain, and its sequence is shown in Additional file 11.

Many second messenger signalling systems possess specialized mechanisms to regulate the synthesis and degradation of signalling molecules [10]. To date, studies characterizing c-di-AMP and its degradation have focused predominantly on bacteria. *Mycoplasmas*, recognized as the smallest and simplest microorganisms, have been found to contain only DHH-DHHA1 domain-containing PDE in *Mycoplasma pneumoniae* and *Mycoplasma bovis* [7, 8]. In this study, we present biochemical evidence that the c-di-AMP binding protein NadD in *M. ovipneumoniae* represents a class of HD domain-containing hydrolases in *Mycoplasmas* that are distinct from those in *Mycoplasma pneumoniae* and *Mycoplasma bovis* and are capable of directly degrading c-di-AMP or pApA to AMP. Under neutral or acidic pH conditions, the phosphodiesterase activity of NadD is relatively weak. Generally, PDEs exhibit the highest activity at neutral pH [7, 36]. However, our findings indicate that NadD has stronger enzymatic activity in a mildly alkaline pH environment, which aligns with research on the phosphodiesterase Pde1 from *Streptococcus pneumoniae* [37]. Moreover, *M. ovipneumoniae* and c-di-AMP infections can enhance the inflammatory response of host cells. Given that the function of NadD is to hydrolyse c-di-AMP, we speculate that its reduced activity under acidic or neutral conditions may lead to decreased hydrolysis of c-di-AMP, resulting in elevated levels of c-di-AMP that persistently induce the inflammatory response in host cells. This finding is supported by our subsequent findings: following the overexpression of NadD in sheep alveolar macrophages, the expression of inflammatory factors induced by *M. ovipneumoniae* or c-di-AMP in these macrophages significantly decreased.

The c-di-AMP-specific PDE is a crucial enzyme in pathogens that regulates c-di-AMP levels. PDEs modulate host immune responses by altering bacterial c-di-AMP concentrations. For example, the pde2 knockout strain of *Streptococcus pneumoniae* secretes increased levels of c-di-AMP when infecting host macrophages. The elevated concentration of c-di-AMP in the cytoplasm of host cells triggers a type I interferon response, leading to the production and secretion of large amounts of IFN-β [22]. Additionally, PDEs can influence the expression of virulence factors in pathogens by regulating c-di-AMP levels, which further impacts the immune response of host cells. The PDE in *Streptococcus pyogenes* affects the processing and maturation of SpeB at the posttranslational level and is essential for the virulence of this bacterium [38]. Our study revealed that the ratio of NadD to DacM dynamically changed as the duration of *M. ovipneumoniae* infection increased, suggesting that these two enzymes may synergistically regulate c-di-AMP levels during the infection process. This modulation may influence the *M. ovipneumoniae*-induced host inflammatory response, thereby affecting the immune response of sheep alveolar macrophages.

*Mycoplasma* is the smallest type of microorganism, and its genetic modification is relatively challenging [39]. Consequently, some researchers have introduced Mycoplasma protein-coding genes into mammalian host cells for expression to investigate their regulation of host cell immune responses [40, 41]. In this context, we constructed a eukaryotic expression plasmid for the NadD protein and transferred it into sheep alveolar macrophages to explore its function. Both fluorescence observations and western blot results demonstrated that the NadD protein can be expressed in sheep alveolar macrophages. Further studies revealed that after *M. ovipneumoniae* infection and c-di-AMP treatment, NadD protein overexpression led to significant decreases in the expression of *IL-1α*, *IL-6*, *TNF-α*, and *IFN-β* induced by *M. ovipneumoniae* or c-di-AMP to varying degrees. These findings suggest that the NadD protein may play a role in inhibiting the inflammatory immune response of host cells during *M. ovipneumoniae* infection. This finding is consistent with research on cyclic dinucleotide PDE (CdnP) in *Group B streptococci* and *Mycobacterium tuberculosis*. Specifically, *Group B Streptococcus* CdnP regulates STING-dependent type I interferon production by degrading c-di-AMP [24]. Similarly, when *Mycobacterium tuberculosis* CdnP infects macrophages, it hydrolyses host-derived c-di-AMP, thereby inhibiting the activation of the STING signalling pathway and the type I interferon response in macrophages, which promotes the survival of *Mycobacterium tuberculosis* within cells [23].

**Figure 6:**
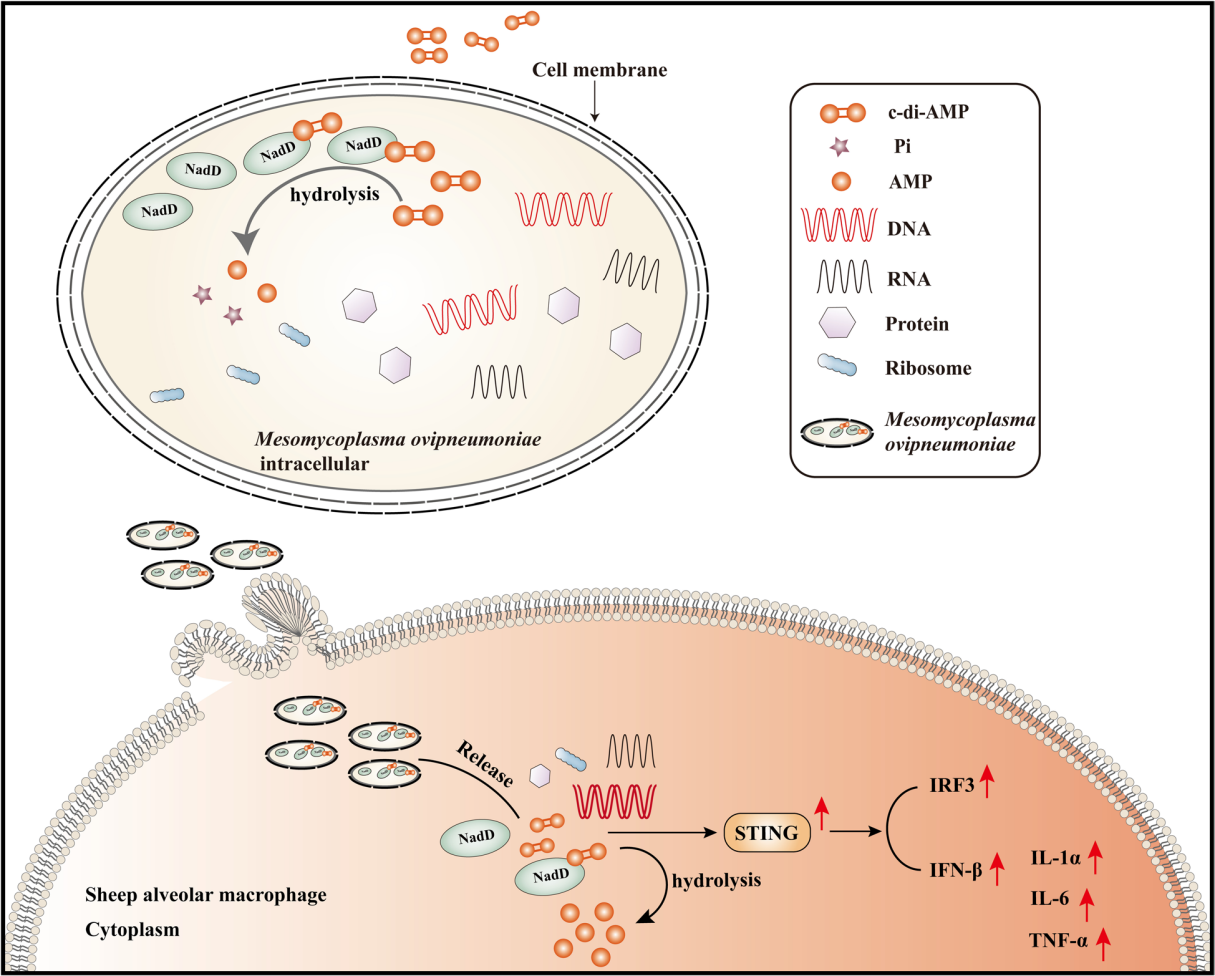
**A schematic diagram of the c-di-AMP molecule and the phosphodiesterase NadD protein from** ***M. ovipneumoniae***. The NadD protein specifically binds to c-di-AMP and exhibits phosphodiesterase activity, which can inhibit the transcription of various inflammatory factors induced by *M. ovipneumoniae*.

In summary, the results of this study indicate that c-di-AMP in *M. ovipneumoniae* can activate the immune response of primary sheep alveolar macrophages. The NadD protein specifically binds to c-di-AMP and exhibits phosphodiesterase activity, which can inhibit the transcription of multiple inflammatory factors induced by *M. ovipneumoniae* (Figure 6). This phenomenon may be associated with the pathogenic process of *M. ovipneumoniae*; however, the specific molecular mechanisms involved require further investigation. Interestingly, research indicates that immune cells cultured at physiological temperatures, which simulate ruminant conditions, exhibit differences in immune responses to bacterial infections compared with those cultured at 37 °C [42]. However, in this study, we selected 37°C as the temperature for in vitro culture of sheep alveolar macrophages, as this temperature is standard for most immunological assays. Additionally, numerous studies on primary sheep cell culture and infection have utilized 37 °C [43–45]. Currently, we have drawn conclusions in this study on the basis of repeated experiments conducted at 37 °C, which can reveal the specific role of NadD in the infection of sheep macrophages by *M. ovipneumoniae*. In the future, further exploration of the immune response of immune cells cultured at physiological temperatures in sheep during *M. ovipneumoniae* infection will more closely align with actual physiological conditions.

## Supplementary Information

**Additional file 1. The primer sequences.**

**Additional file 2. Evaluation of the model of primary sheep alveolar macrophages infected with** ***M. ovipneumoniae***. (**A**) Identification of isolated primary sheep alveolar macrophages using CD14 immunofluorescence. (**B**) Detection of the *M. ovipneumoniae* marker gene Hsp70 and the housekeeping gene GAPDH in sheep alveolar macrophages at 0 h, 6 h, 12 h, and 24 h post-*M. ovipneumoniae* infection. (**C**) Higher magnification images showing the distribution details of *M. ovipneumoniae* in sheep alveolar macrophages and the morphology of its inclusions. (**D**–**I)** HPLC analysis of c-di-AMP content in sheep alveolar macrophages at 0 h, 3 h, 6 h, 12 h, and 24 h following *M. ovipneumoniae* infection.

**Additional file 3. The list of genes and pathways that are similarly induced by** ***M. ovipneumoniae*** **and c-di-AMP**.

**Additional file 4. The list of the 30 unique proteins identified in the c-di-AMP group, as well as 246 proteins that are commonly expressed at higher levels in the c-di-AMP group.**

**Additional file 5. Functional enrichment analysis of c-di-AMP-binding proteins**. (**A**) GO enrichment analysis of c-di-AMP-binding proteins. (B) KEGG enrichment analysis of c-di-AMP-binding proteins.

**Additional file 6. Phylogenetic tree analysis of the NadD protein.**

**Additional file 7. Homology comparison of the C-terminal domain of the NadD protein.**

**Additional file 8. HPLC analysis of c-di-AMP hydrolysis by NadD at various pH values.**

**Additional file 9. HPLC analysis of NadD-mediated hydrolysis of c-di-AMP in the presence of different metal ions.**

**Additional file 10. Distribution of the NadD gene across various strains of** ***M. ovipneumoniae***.

**Additional file 11. The NadD gene sequence.**

## Data Availability

All the data generated or analysed during this study are included in this article and its additional files.
