# Supplementary material for: The c-di-AMP binding protein NadD from Mesomycoplasma ovipneumoniae functions as a phosphodiesterase that inhibits host inflammatory responses
Source: Vet Res. 2026 Jan 9;57:29. doi: 10.1186/s13567-025-01707-5 (PMC12879401; doi:10.1186/s13567-025-01707-5)
Supplement: Supplementary file 1 — Additional file 1. The primer sequences. [file 13567_2025_1707_MOESM1_ESM.docx]

**Additional file 1 The primer sequences.**

| Gene | Forward primer (5’-3’) | Reverse primer (5’-3’) |
| --- | --- | --- |
| *ADORA3* | CCAACTCATTGTCACTGTGTCCTG | TGTCGGTGATGGCTCTTCTGTC |
| *CCR2* | GAAGAACCCACCACCAGTTATGAC | GTAGAGCGGCGGCAGGAG |
| *CSF1R* | ACTGGTGCGGATTCAAGGAGAG | TTGTCACGGAAGTCGGATTGTTG |
| *CXCL8* | GCTGGCTGTTGCTCTCTTGG | GGGTGGAAAGGTGTGGAATGTG |
| *GAPDH* | GGAGAAACCTGCCAAGTATGATGAG | GAGTGAGTGTCGCTGTTGAAGTC |
| *IFIT3* | GCTGGACTGTGAGGAAGGATGG | ATTCTGGGTTGTTGGGTTTCTCTTC |
| *IFN-β* | ACCTCCTGTGGCAGTTACCTTC | GCTGTGCTTGCTTCATCTCCTC |
| *IL-1β* | ATGGCTTGCTACAGTGATGAGAATG | GAGCCGAGGTCCAGGTGTTG |
| *IL-1α* | CAAGGAGAATGTGGTGATGGTGAC | TGCTGATCTGGGCTTGATGATTTC |
| *IL-27* | CCTTCCTGAAGAGCATGAGAATGAG | GTCCGAGACAAGACAAGTTCCAAG |
| *IL-6* | GTCTAATAACCACTCCAGCCACAC | TAACCTTTGCGTTCTTTACCCACTC |
| *IRF3* | GTGTTGCGTTTAGCGGAGGAC | CTGCCATTGTCTTGAGCGGTATC |
| *ISG15* | CAGCAGCTCCTATGAGGTCCAG | CTTGCACACGCTCCCTTTGG |
| *MX1* | ACCAGCCACACGACATTGAATATC | TTAGCAGGGACCACCACCAAG |
| *PIK3IP1* | AACGGGTGCGGGTGAACTC | TGTAGGTGTAGCCAAGGACGATTC |
| *SMAD6* | AGCATCTTCTACGACCTACCTCAG | GCCCACACGCCGTCAGG |
| *STING* | CAACTGCCGCCTCATTGTCTAC | TTCCCAGATCCAGAACCTTCCAG |
| *TNF-α* | CAACGGCGTGGAGCTGAAAG | TGAAGAGGACCTGCGAGTAGATG |
