# Supplementary figures and images for: The c-di-AMP binding protein NadD from Mesomycoplasma ovipneumoniae functions as a phosphodiesterase that inhibits host inflammatory responses
Source: Vet Res. 2026 Jan 9;57:29. doi: 10.1186/s13567-025-01707-5 (PMC12879401; doi:10.1186/s13567-025-01707-5)

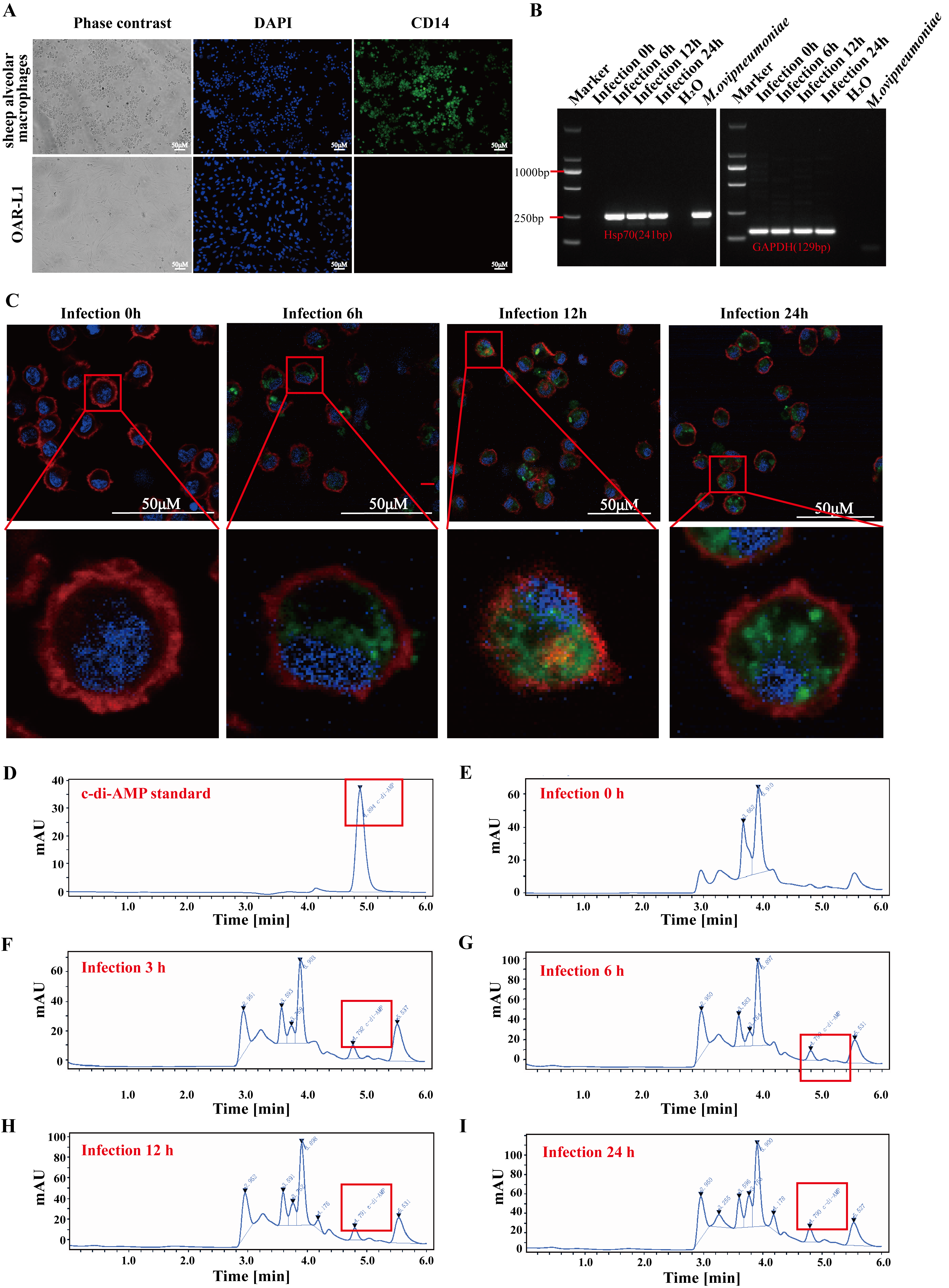

Supplement: Supplementary file 2 — Additional file 2. Evaluation of the model of primary sheep alveolar macrophages infected with M. ovipneumoniae. (A) Identification of isolated primary sheep alveolar macrophages using CD14 immunofluorescence. (B) Detection of the M. ovipneumoniae marker gene Hsp70 and the housekeeping gene GAPDH in sheep alveolar macrophages at 0 h, 6 h, 12 h, and 24 h post-M. ovipneumoniae infection. (C) Higher magnification images showing the distribution details of M. ovipneumoniae in sheep alveolar macrophages and the morphology of its inclusions. (D–I) HPLC analysis of c-di-AMP content in sheep alveolar macrophages at 0 h, 3 h, 6 h, 12 h, and 24 h following M. ovipneumoniae infection. [file 13567_2025_1707_MOESM2_ESM.tif]

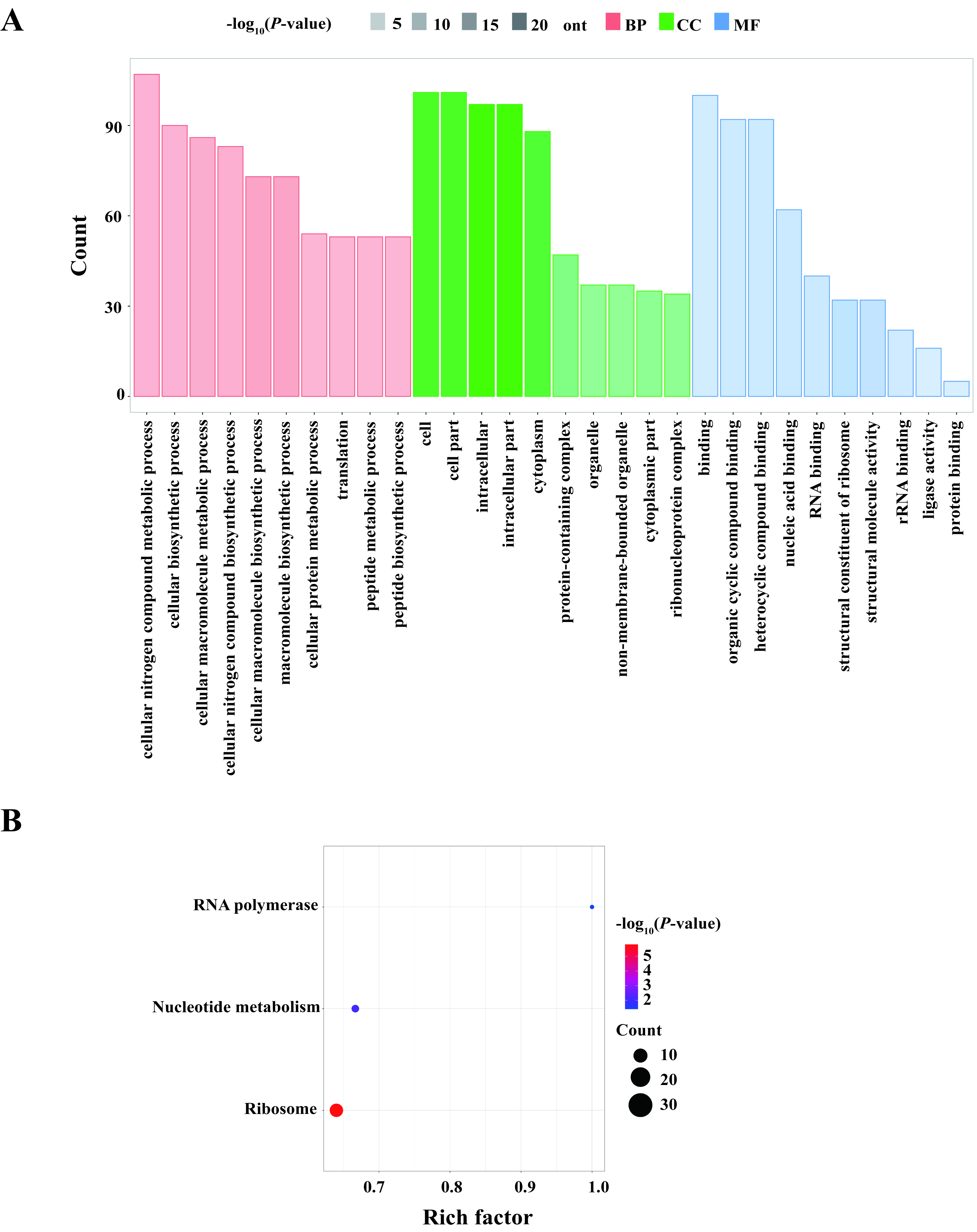

Supplement: Supplementary file 5 — Additional file 5. Functional enrichment analysis of c-di-AMP-binding proteins. (A) GO enrichment analysis of c-di-AMP-binding proteins. (B) KEGG enrichment analysis of c-di-AMP-binding proteins. [file 13567_2025_1707_MOESM5_ESM.tif]

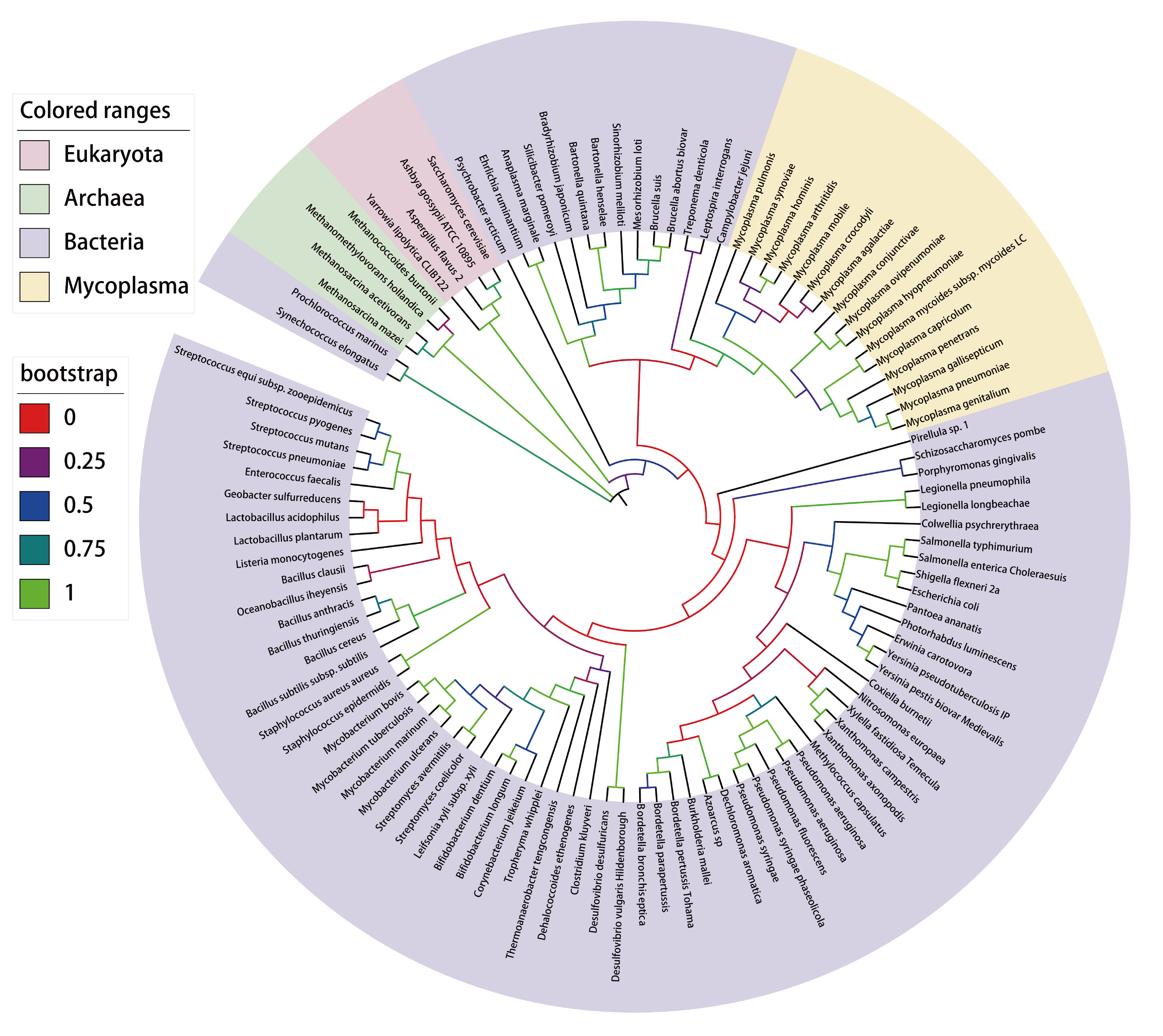

Supplement: Supplementary file 6 — Additional file 6. Phylogenetic tree analysis of the NadD protein. [file 13567_2025_1707_MOESM6_ESM.tif]

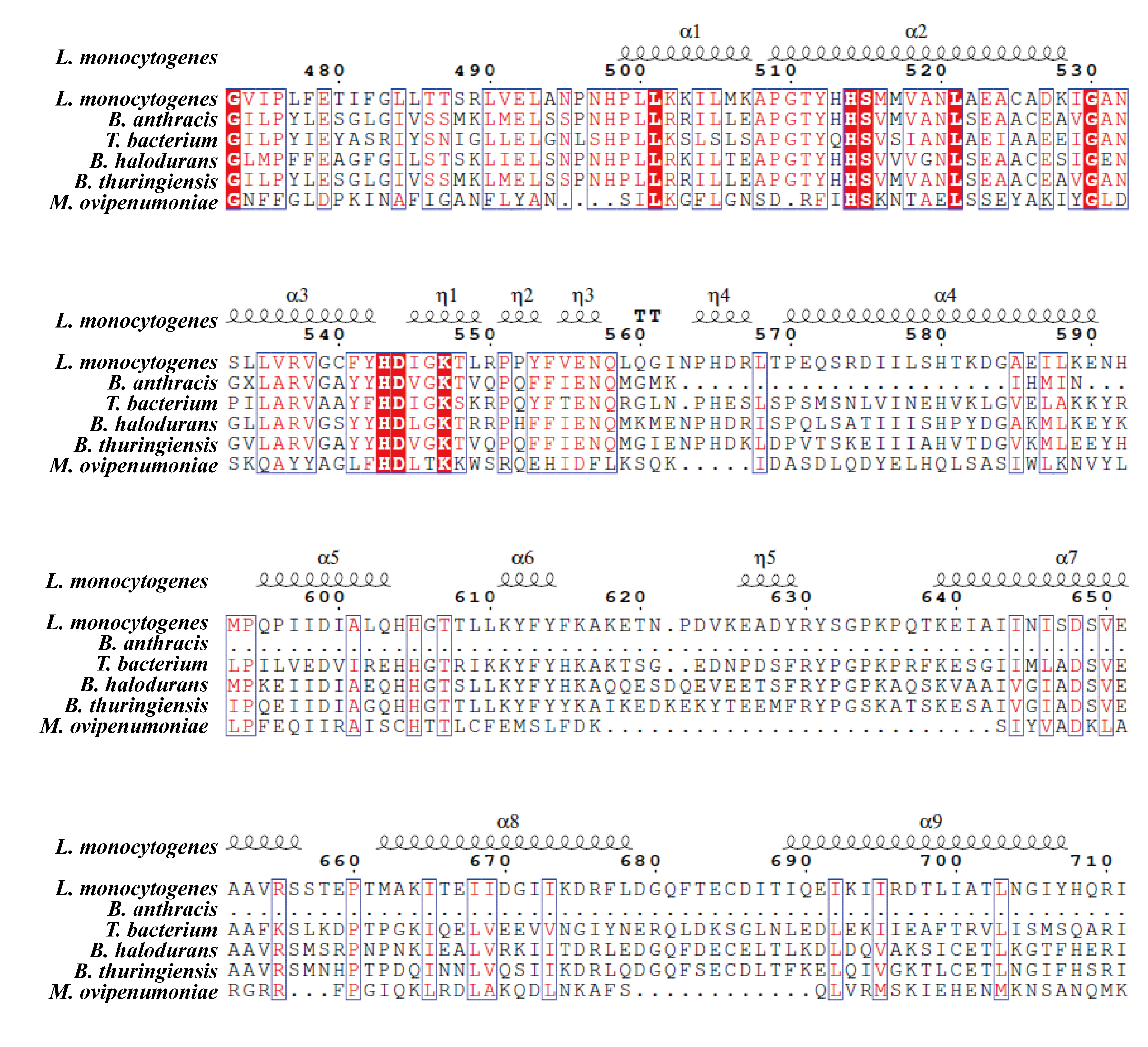

Supplement: Supplementary file 7 — Additional file 7. Homology comparison of the C-terminal domain of the NadD protein. [file 13567_2025_1707_MOESM7_ESM.tif]

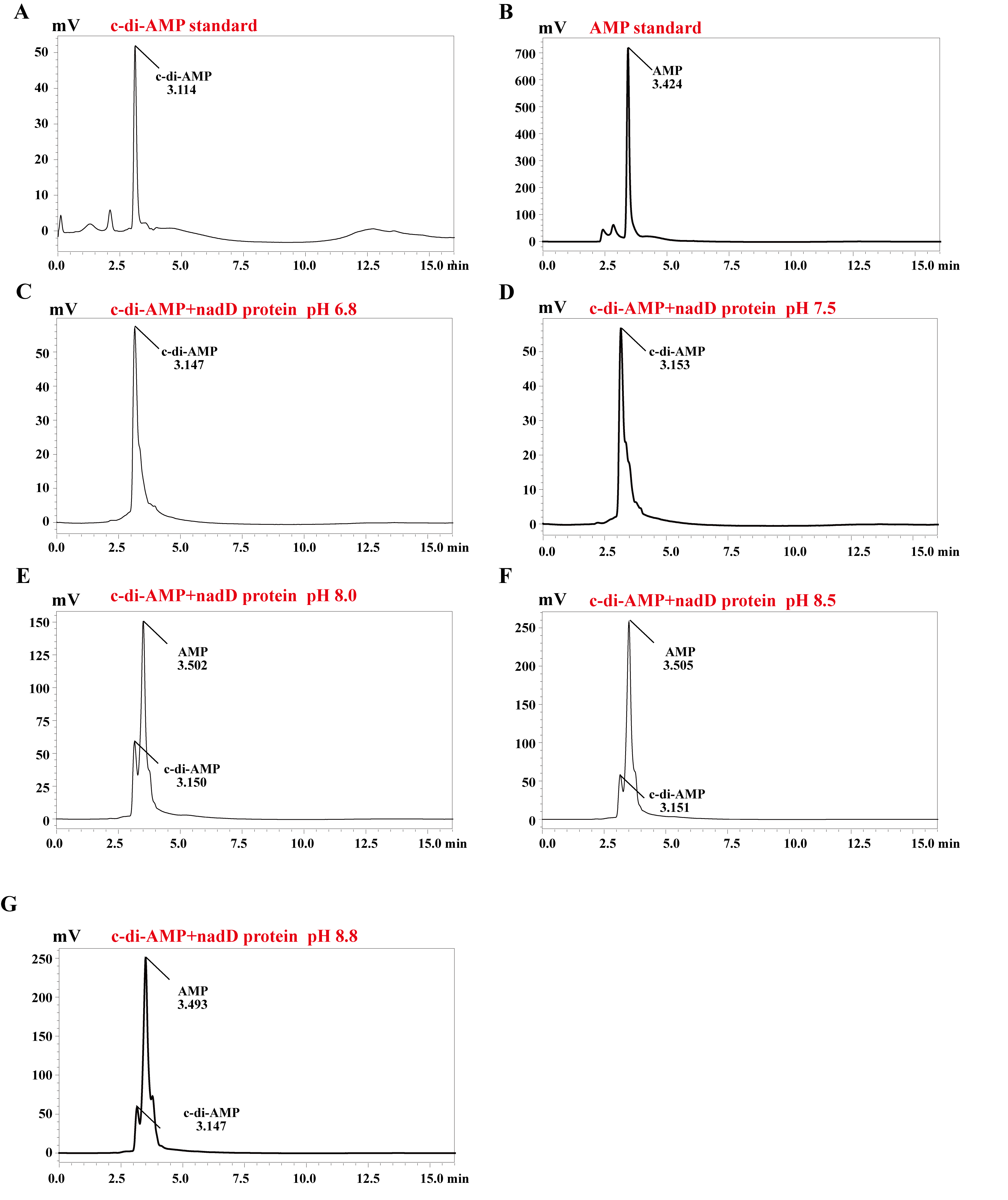

Supplement: Supplementary file 8 — Additional file 8. HPLC analysis of c-di-AMP hydrolysis by NadD at various pH values. [file 13567_2025_1707_MOESM8_ESM.tif]

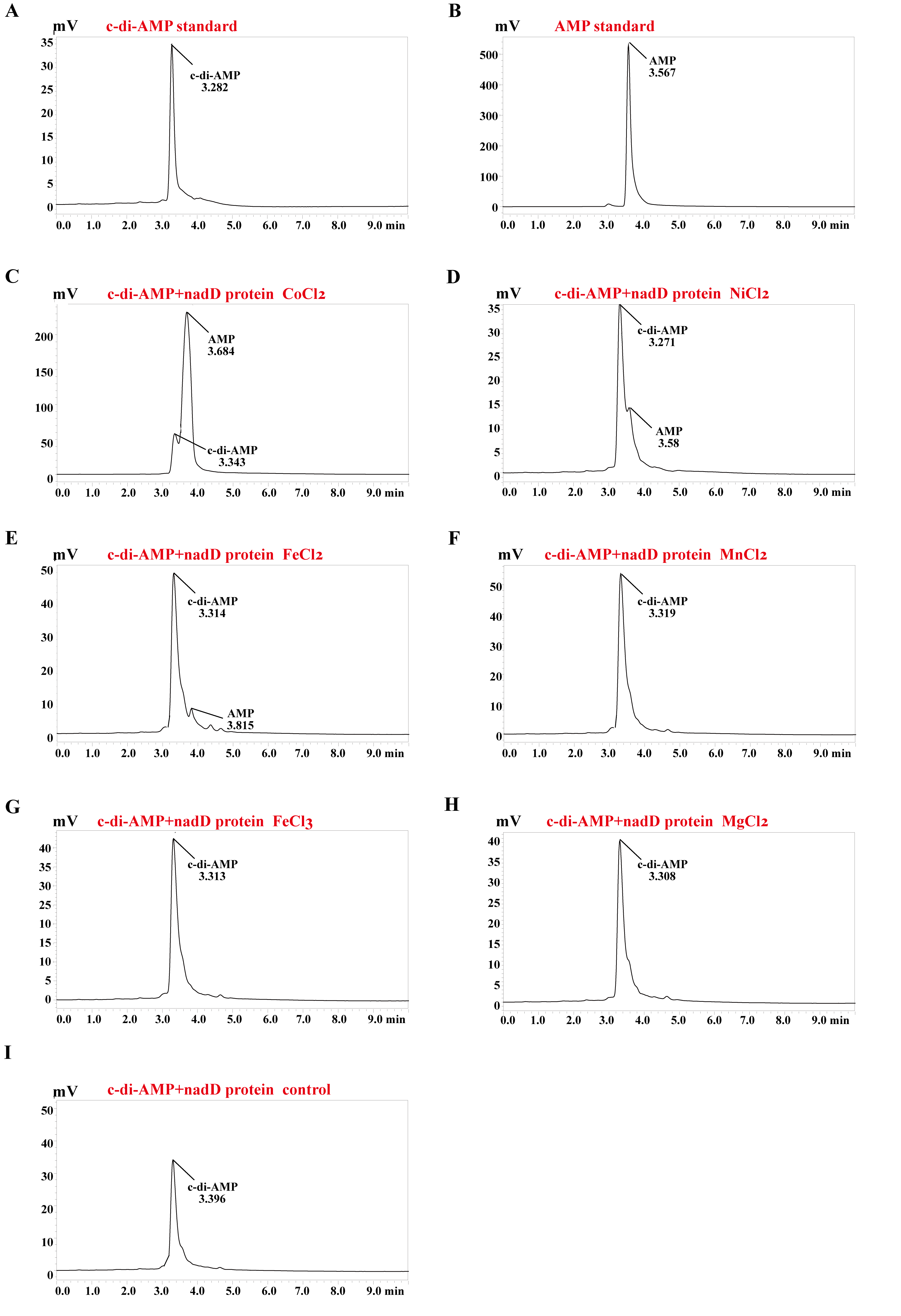

Supplement: Supplementary file 9 — Additional file 9. HPLC analysis of NadD-mediated hydrolysis of c-di-AMP in the presence of different metal ions. [file 13567_2025_1707_MOESM9_ESM.tif]
