# Supplementary material for: The c-di-AMP binding protein NadD from Mesomycoplasma ovipneumoniae functions as a phosphodiesterase that inhibits host inflammatory responses
Source: Vet Res. 2026 Jan 9;57:29. doi: 10.1186/s13567-025-01707-5 (PMC12879401; doi:10.1186/s13567-025-01707-5)
Supplement: Supplementary file 11 — Additional file 11. The NadD gene sequence. [file 13567_2025_1707_MOESM11_ESM.docx]

*NadD* nucleotide sequence from *Mesomycoplasma ovipneumoniae* strain Y98

Atgaaattagaaaaaattgcaatttatggcggttcatttaaccccgttcataaagctcatatacaaattgcaaaaaaagcaatcgattttttaaatttagatatgcttttttttgtgccaaattacataaatccactaaaaaacaataaagaaaacaacattgatcccgcatttcgttttgaaatgctaaaattagtgcaaattgaaaaaacacaagtttgtgattatgaaattaaagccaaaaaaattagctacacaattgaaacagtaaattatttcaagcaaaaatacgcaggtgccaaactttttttaataataggttctgataatcttgccagttttaaaatgtggaaaaattacaaggaaattcttgaaaaagtccaacttgttgtttttaggcgcaaaaattatcctgatttaggaaatgttaaacgttataatgccttaattttgccaacccagttgcctaattttagctcttcagaaataagaaatggaaacttttttggccttgatccaaaaataaatgcctttattggcgctaattttttatatgctaattcaattcttaaaggttttcttggtaattctgaccgatttatccattcaaaaaataccgctgaactctcaagcgagtatgctaaaatttatggactagattcaaaacaagcttattatgctggcttatttcatgatttaaccaaaaaatgaagcaggcaagaacatattgattttcttaaatcccaaaaaattgatgctagcgatcttcaagactatgaacttcaccaactttcagcttcaatttgattaaagaatgtttatttattaccttttgagcaaataatccgcgctatttcttgtcatacaaccctttgttttgaaatgtcattatttgacaaatcaatttatgttgccgataaattagctcgtggccgtcgttttccaggtattcaaaaattacgcgacttagcaaaacaagatttaaataaagcctttagccaacttgtaagaatgtccaaaattgagcatgaaaatatgaaaaactcagccaatcaaatgaaactatatgaaaaacatcaaaattaa
